# Cardiometabolic risk in children and adolescents with obesity: a position paper of the Italian Society for Pediatric Endocrinology and Diabetology

**DOI:** 10.1186/s13052-024-01767-x

**Published:** 2024-10-08

**Authors:** Giuliana Valerio, Procolo Di Bonito, Valeria Calcaterra, Valentino Cherubini, Domenico Corica, Luisa De Sanctis, Anna Di Sessa, Maria Felicia Faienza, Elena Fornari, Lorenzo Iughetti, Maria Rosaria Licenziati, Melania Manco, Emanuele Miraglia del Giudice, Anita Morandi, Mariacarolina Salerno, Maria Elisabeth Street, Giuseppina Rosaria Umano, Malgorzata Wasniewska, Claudio Maffeis

**Affiliations:** 1 Department of Medical, Movement and Wellbeing Sciences, University of Napoli “Parthenope”, Napoli, 80133 Italy; 2 Department of Internal Medicine, “S. Maria delle Grazie” Hospital, Pozzuoli, 80078 Italy; 3 Pediatric Department, “V. Buzzi” Children’s Hospital, 20154 Milano, Italy; 4 Department of Internal Medicine, University of Pavia, Pavia, 27100 Italy; 5 Department of Women’s and Children’s Health, Azienda Ospedaliero-Universitaria delle Marche, Ospedali Riuniti di Ancona, “G. Salesi Hospital,”, Ancona, Italy; 6 Department of Human Pathology in Adulthood and Childhood, University of Messina, Messina, 98122 Italy; 7 Department of Public Health and Pediatric Sciences, University of Turin, Turin, 10126 Italy; 8 Department of Woman, Child and of General and Specialized Surgery, Università degli Studi della Campania “Luigi Vanvitelli”, Napoli, 80138 Italy; 9 Pediatric Unit, Department of Precision and Regenerative Medicine and Ionian Area, University of Bari “Aldo Moro”, Bari, 70124 Italy; 10 Department of Surgery, Dentistry, Pediatrics and Gynecology, Section of Pediatric Diabetes and Metabolism, University and Azienda Ospedaliera Universitaria Integrata of Verona, Verona, 37126 Italy; 11 Paediatric Unit Department of Medical and Surgical Sciences of Mothers, Children and Adults, University of Modena and Reggio Emilia, Modena, 41121 Italy; 12 Neuro-Endocrine Diseases and Obesity Unit, Department of Neurosciences, Santobono- Pausilipon Children’s Hospital, Naples, 80129 Italy; 13 Preventive and Predictive Medicine Unit, Bambino Gesù Children’s Hospital, IRCCS, Rome, 00165 Italy; 14 Department of Translational Medical Science, University of Naples “Federico II”, Napoli, 80131 Italy; 15 Department of Medicine and Surgery, University of Parma, Parma, 43125 Italy

**Keywords:** Cardiometabolic risk, Dyslipidemia, Hypertension, Kidney disease, Left ventricular hypertrophy, Metabolic dysfunction-associated steatotic liver disease, Obstructive sleep apnea, Pediatric obesity, Physical inactivity, Polycystic ovary syndrome

## Abstract

Despite the implementation of preventive measures to counteract the obesity epidemics, the prevalence of childhood obesity is still alarming all over the world. Childhood obesity is the most common risk factor for both cardiovascular and metabolic diseases. In fact, an earlier onset of obesity can cause a greater risk of adiposity tracking across the lifespan and consequently a longer exposure to cardiometabolic risk factors. Accumulating evidence provided by prospective and intervention studies demonstrated the link between pediatric obesity and selected subclinical signs of cardiovascular damage (atherosclerosis and left ventricular hypertrophy), or fatal and not fatal cardiovascular events as early as 40 years of age.

The numerous guidelines and scientific documents published in the last years demonstrate the relevance of assessing cardiometabolic risk factors in children and adolescents with OB.

This Position paper, released by experts of the “Childhood Obesity study group” within the Italian Society for Pediatric Endocrinology and Diabetology, aims to review the assessment of cardiometabolic risk factors and comorbidities in children and adolescents with OW/OB on the light of the most recent scientific evidence.

The main recommendations are: (a) early detection of comorbidities, including hypertension, dyslipidemia, prediabetes/type 2 diabetes, metabolic dysfunction-associated steatotic liver disease, polycystic ovary syndrome, inactivity, obstructive sleep apnea and decline in kidney function; *(b)* weight loss treatment, which is associated with a reduction of all cardiometabolic risk factors; *(c)* specific treatment of comorbidities, through lifestyle modifications or pharmacological treatment added to lifestyle for suitable individuals; *d).* monitoring comorbidities for mitigating future morbidity and mortality.

**Supplementary Information:**

The online version contains supplementary material available at 10.1186/s13052-024-01767-x.

## Introduction

Despite the slight reduction in the prevalence of childhood obesity (OB) obtained through sustained preventive programs in the last years, the ongoing surveillance systems show that the prevalence of OB is still alarming all over the world. The last Italian 2019 report underlines the presence of overweight (OW) in 20.4% children, OB in 9.4% and severe OB in 2.4% [1]. There are several compelling evidences that childhood OB is the most common risk factor for both cardiovascular (CV) and metabolic diseases [2].

Two conditions are worthy of particular attention: OB onset under 5 years of age and severe OB. An earlier onset of OB can cause a greater lifetime exposure to cardiometabolic risk factors (CMRFs) [3]. Indeed, a systematic review with meta-analysis reported a medium/strong tracking of adiposity across the lifespan and an association between childhood OB and selected adult CV risk factors [4]. Moreover, a higher prevalence of CMRFs, such as insulin resistance (IR), hypertension (HTN), dyslipidemia, and prediabetes, has been reported in children with severe OB compared to milder forms [5, 6].

Since CV disease in childhood is rare, it is difficult to demonstrate the value of relative contribute of each single CMRF in childhood as compared to adulthood, when subclinical and clinical disease are prevalent. However, recent longitudinal studies demonstrated that some risk factors in childhood, such as HTN and dyslipidemia, are associated with subclinical atherosclerosis and left ventricular hypertrophy (LVH) in adult life. Of note, Franks et al. have shown that children in the highest quartile of body mass index (BMI) had more than double the rate of premature death (including CV disease) than those in the lowest [7]. According to a recent systematic review, robust evidence linked hypertension, hyperlipidemia, and risk factor clustering with subclinical CV disease, while limited evidence linked these factors with adult clinical CVD [8]. In this context of uncertain results, an important prospective study was performed on the behalf of the International Childhood Cardiovascular Cohort (i3C) Consortium [9]. This study, that included seven cohorts in Australia, Finland, and the United States, found that childhood risk factors at the age of 3–19 years were associated individually and in combination with fatal and not fatal CV events as early as 40 years of age.

Based on these premises, children with OW/OB should be monitored closely and screened periodically for obesity-related CV risk factors. The numerous guidelines and scientific documents published in the last years demonstrate the relevance of assessing CMRFs in children and adolescents with OB. A recent scoping review of 30 international and national guidelines on the management of pediatric OB found that most of them (76%) had some guidance on screening for metabolic complications, namely, HTN, type 2 diabetes (T2D), dyslipidemia, and non-alcoholic fatty liver disease (NAFLD) [10]. The Authors reported significant variations between guidelines in the extent and details of recommendations, such as BMI thresholds, age of onset, frequency, and screening tests. Only few months later, the American Academy of Pediatrics (AAP) released a technical report that provided evidence-based information for the management of OB comorbidities [11].

In addition, as new studies are reviewed and graded, several guidelines specifically dedicated to the assessment and management of childhood HTN, diabetes, lipid and fatty liver disease (FLD) have been updated [12–16].

Thus, experts of the “Childhood Obesity study group” of the Italian Society for Pediatric Endocrinology and Diabetology decided to update the Italian Consensus position statement on diagnosis, treatment and prevention of pediatric obesity released in 2018 [17] with the aim to review the assessment of CMRFs in children and adolescents with OW/OB on the light of the most recent scientific evidences.

This position statement follows the publication of an updated consensus document on the treatment of pediatric obesity recently released by the same group of experts [18], to which any interested reader should refer to for more details on lifestyle intervention, drugs, and bariatric surgery.

### Adiposity measures and cardiometabolic risk

Obesity is as a state of excessive adipose tissue, that is strongly associated with an increased prevalence of CMRFs. Since direct measurement of body fat is rarely available in the clinical practice, the weight-to-length ratio (< 2 years) or the BMI (*≥* 2 years), which measure excess weight, and not body fat, are the most frequently used tool to diagnose and classify OW or OB. Despite this limit, stratification of weight categories based on age- and sex-specific BMI percentiles showed a higher prevalence of abnormal levels of cardiometabolic variables with greater severity of OB [19]. As BMI is strictly dependent on sex and age, several BMI metrics can be used, i.e. BMI Z-score, BMI percentile, percent of the BMI 95th percentile (%BMIp95), or percent of the BMI median (%BMIp50). It was found that all these metrics significantly correlated with adiposity, visceral adiposity and CMRFs in children and adolescents, but %BMIp95 and %BMIp50 were the indices most strongly correlated with measures of adiposity [20]. However, there is emerging evidence to suggest that central adiposity is increasing at a faster rate than BMI also in childhood [21]. Therefore, other tools, such as waist circumference (WC), have been proposed as surrogate of abdominal fat content. Specifically, WC may be useful in addition to BMI, in the stratification of CMR in childhood with OW/OB [22]. For instance, it has been demonstrated that youths with high WC values were more likely to have elevated CMRFs compared with those with low WC values, within a given BMI category [23]. Unfortunately, the use of WC in the clinical practice is undermined by the lack of cut points and widely accepted reference data for children and/or adolescents (only few country-specific references are available). Therefore, the waist-to-height ratio (WHtR) is an alternative simple and quick tool to screen for abdominal fat and CMR in children. Compared to BMI and WC, WHtR is much less variable with age, and a single cut-off can be used for stratification across age range. On the contrary, different cut-offs should be considered for East and Southeast Asian regions and Latin American regions [24]. A WHtR cut-off value > 0.50 has been demonstrated to predict central OB with high accuracy [25] and to be related to worse metabolic profile in children and adolescents from the general population [26]. Regarding children and adolescents with OB, a cut-off ≥ 0.60 has been proposed to predict CMR [27–29]; furthermore, a reduction of this index over time was associated with an improvement in the CMR profile [27].

In the light of current knowledge, WHtR did not have significantly better screening power than BMI and WC in most traditional CMRFs, except for elevated triglycerides (TG) when compared with BMI and high metabolic risk score when compared with WC [30].

#### Panel recommendation

BMI is essential for the screening of children > 5 years of age with OW, OB and severe OB as well as for the stratification of CMR. The cut-offs for definition are shown in Table 1. The WHtR measure may provide additional support to suspect CMR. In youths with OB, the combination between BMI and WHtR can be useful to monitor overall and abdominal adiposity changes during weight management programs.

**Table 1:** Cardiometabolic risk factors

	Cut-offs
**Weight excess**	
***Overall adiposity*** ^a, b,c^	
**0–2 years**	
At risk of overweight	Weight-to-length ratio Z-score > + 1 and <=+2
Overweight	Weight-to-length ratio Z-score > + 2 and <=+3
Obesity	Weight-to-length ratio Z-score > + 3
**2–5 years**	
At risk of overweight	BMI Z-score > + 1 and <=+2
Overweight	BMI Z-score > + 2 and <=+3
Obesity	BMI Z-score > + 3
**5–18 years**	
Overweight	BMI Z-score > + 1 and <=+2
Obesity	BMI Z-score > + 2 and <=+3
Severe Obesity	BMI Z-score > + 3
***Visceral adiposity***	
WHtR	≥ 0.60
***High blood pressure*** ^d^	
Elevated BP	Systolic and/or diastolic BP (mmHg) ≥ 90th < 95th pc. for age, sex, and height
Hypertension	Systolic and/or diastolic BP (mmHg) ≥ 95th pc. for age, sex, and height (under 16 years) ^e^
	Systolic and/or diastolic BP (mmHg) ≥ 135/80 (16–18 years)
***Glucose dysmetabolism***	
***Prediabetes***	
Impaired fasting glucose	Fasting glucose ≥ 100 < 126 mg/dL
High HbA1c	HbA1c ≥ 5.7 < 6.5% (≥ 39 < 48 mmol/mol)
Impaired glucose tolerance	2-h glucose during oral glucose tolerance test ≥ 140 < 200 mg/dL
***Diabetes***	Fasting glucose ≥ 126 mg/dL or HbA1c ≥ 6.5% (≥ 48 mmol/mol) or 2-h glucose ≥ 200 mg/dL ^f^
***Dyslipidemia***	
High LDL-Cholesterol	LDL- Cholesterol calculated ≥ 130 mg/dL
High cholesterol	Total cholesterol ≥ 200 mg/dL
High triglycerides	Triglycerides ≥ 100 mg/dL (< 10 years) or TG ≥ 130 mg/dL (≥ 10years)
Low HDL-Cholesterol	HDL- Cholesterol <40 mg/dL
***Physical inactivity***	
Physical activity	< 60 min/day
Sedentary behavior	≥ 2 h/day

^a^ Weight-to-length or BMI z-scores of + 1, + 2 and + 3, approximate the 85th, 97th and 99th pcs, respectively

^b^Normative BMI tables are available from: https://www.who.int/tools/child-growth-standards/standards/weight-for-length-height (0–2 years); https://www.who.int/toolkits/child-growth-standards/standards/body-mass-index-for-age-bmi-for-age (2–5 years); https://www.who.int/tools/growth-reference-data-for-5to19-years (5–18 years)

^c^ software to calculate weight-to-length or BMI percentile or Z-scores is available at http://www.weboriented.it/gh4/downloads/index.php

^d^ Normative systolic and diastolic BP tables arranged by age, sex, and height (and height percentile) [15]

^e^ Confirm on two occasions within 2–3 weeks

^f^ Confirm on two occasions

Abbreviations: BMI: body mass index; BP: blood pressure; HbA1c: glycosylated hemoglobin A1c; HDL: high density lipoprotein; LDL: low density lipoprotein.; pc: percentile

### Hypertension

High blood pressure (BP) is frequently reported in children with OW/OB. HTN was found respectively in 5.0% of OW and 15.3% of OB youths compared to 1.9% normal-weight children [31]. The standard procedure for the diagnosis of HTN has been based on systolic and/or diastolic BP values ​​≥95th percentile for age, sex, and height provided by the Fourth report on the diagnosis, evaluation, and treatment of high BP in children and adolescents [32]. The former reference tables were replaced by those proposed by the AAP in 2017 after the exclusion of individuals with OW/OB [12]. Furthermore, the AAP proposed the use of table percentiles for age < 13 years a cut-off of BP ≥ 130/80 mmHg over 13 years of age, as it was proposed in adults. In 2020 the Canadian guidelines accepted the new AAP tables of BP, but in view of a simplification, proposed static cut-offs in children (< 12 years) ≥ 120/80 mmHg and ≥ 130/85 in adolescents (age ≥ 12 years), respectively [33].

In 2022 a Panel of experts of the European Society of Cardiology (ESC) agreed upon the new normative tables released by the AAP, but did not support the use of fixed cut off at 13 years for several reasons: (1) incomplete pubertal maturation at this age; (2) systolic BP levels rarely exceed the normal adult cut-point between 13 and 16 years, especially in tall boys; (3) the power in the identification of early hypertensive organ damage is still unknown. Therefore, it was suggested to use percentiles for age ≤ 16 years, and a fixed cut-off of 130/85 mmHg in the transition age of 16–18 years [13].

Italian cross-sectional studies demonstrated the advantage of the 2017 AAP tables over the 2004 Fourth Report tables in young people with OW/OB. About 11% youths who were discordantly classified (non-hypertensive according to the 2004 tables but hypertensive according to the 2017 tables) showed higher BP levels and abnormal CMRFs [34]. Another comparative study underlined the advantage of using the 2017 percentiles of BP, as recommended by ESC consensus document, against the Canadian fixed cutoffs in terms of primary CV prevention [35].

The screening should be performed in all children and adolescents with OW/OB starting from 2 years of age and repeated at each visit. Several guidelines recommend that HTN should be confirmed on 3 different occasions (moderate level of evidence). Since this procedure exposes youths with OW/OB at high risk of drop-out [36–38], we agree with the ESC consensus document that a second measure of BP within 2–3 weeks from the first finding may be sufficient for confirmation of HTN.

Ambulatory BP monitoring can be used in selected cases (suspected white coat, secondary HTN, diabetes, monitoring of antihypertensive therapy and clinical trials) in specialized centers. However, the lack of the normative tables, which are representative of the young general population (those in use are derived from a German population) limits the implementation of this method in clinical practice [39].

Echocardiographic evaluation may be undertaken at presentation of HTN to identify LVH as an index of target damage, and to address the need and monitoring of drug therapy (see next paragraph).

The best therapeutic approach either in case of elevated BP or HTN corresponds to the multidisciplinary treatment of OB. The initial approach is based on hypocaloric diet, reduction of sodium intake and screen time and increase of moderate/vigorous physical activity (PA). If HTN persists after 6 months, pharmacologic treatment can be started with drugs licensed for use in children, in the presence cardiac or renal damage or failure of non-pharmacological therapy. If HTN Stage 2 is confirmed in the second visit the patient should start therapy immediately or be sent to a specialist center. Antihypertensive agents licensed for use in children include angiotensin converting enzyme inhibitors, angiotensin receptor blockers, dihydropyridine calcium channel blocker [12, 13]. Diuretics or beta blockers are not suggested in children with OW/OB, except in specific conditions, as they may alter glucose metabolism and reduce energy expenditure.

#### Panel recommendation

In agreement with the ESC Consensus document for the diagnosis of HTN in children or adolescents with OW/OB, this Panel suggests the use of the cut-offs of BP by sex, age, and height of the AAP guidelines to define elevated BP (≥ 90th < 95th percentile), Stage 1 HTN (≥ 95th percentile) and Stage 2 HTN (BP ≥ 95th percentile + 12 mm Hg) (Table 1). Only two measurements (within 2–3 weeks) are sufficient to confirm HTN.

### Dyslipidemia

The association between OB and dyslipidemia is predictive of fatal and non-fatal CV events in adult life. Translational, epidemiological and clinical studies have clearly demonstrated the strong link between visceral and ectopic fat and the development of atherogenic dyslipidemia, and atherosclerotic lesions have been proven since pediatric age [40]. Dyslipidemia in OB is mainly driven by the effects of IR and pro-inflammatory adipokines, but it also depends on individual factors [41].

Although it still remains underestimated, the diagnosis of dyslipidemia is increasing both in adulthood and in childhood, due to the worsening in OB prevalence but also to the growing medical attention towards CMRFs in patients of any age [42].

Lipid abnormalities in children and adolescents with OB include elevated serum triglycerides (TG) and non-high-density cholesterol (non-HDL-C) levels and low HDL-C levels. Low density lipoprotein-cholesterol (LDL-C) levels are frequently normal or slightly elevated, but an increase of small dense LDL-C has been demonstrated [43]. According to a recent systematic review and meta-analysis the prevalence of high TG in children and adolescent with OW or OB, is respectively 12.6% and 19.2%, the prevalence of low HDL-C is 15.7% and 20.3%, while elevated LDL-C has been reported in 7.5% and 12.2% [44].

The appropriate age for screening dyslipidemia is still debated. The guidelines from NHLBI, BMJ Best Practice, and the Society for Adolescent Health and Medicine report two windows by age for universal lipid screening in youth, regardless of BMI status: 9–11 years and 17–21 years [45]. However, it is strongly recommended to anticipate lipid screening between 2 and 8 years of age in children with OB, which represents itself a CMRF. The presence of family history of dyslipidemia or premature CV disease, or moderate- or high-risk medical conditions are additional criteria for earlier screening [17, 46]. Most guidelines recommend the fasting lipid profile (TG, total cholesterol, HDL-C and calculated LDL-C) to detect dyslipidemia in children with OB [10]. The importance of lipoprotein (a) dosage in the global estimate of CV risk is also emerging, especially in the context of familial hypercholesterolaemia [47, 48]. Lipoprotein(a) level is genetically determined. It has been demonstrated that concentrations of lipoprotein(a) are independent of BMI, age and sex during childhood [49]. Therefore, the dosage of this lipoprotein is useful for identifying children at high CV risk, independent of obesity.

A total cholesterol *≥* 200 mg/dl, an LDL-C *≥* 130 mg/dl and a HDL-C < 40 mg/dl are the cut-off reference values to define an abnormal lipid profile according to the Expert Panel on Integrated Guidelines for Cardiovascular Health and Risk Reduction in Children and Adolescents [46]. For TG, the cut-off differs according to age: *≥*100 mg/dl up to 9 years and *≥* 130 mg/dl above 10 years of age (Table 1). Consistent evidence unequivocally establishes that the atherosclerotic risk linked to dyslipidemia is a cumulative risk, determined by both the concentration of circulating LDL-C and other ApoB-containing lipoproteins, and the total duration of exposure to these lipoproteins. This evidence justifies the importance of increased awareness, early identification, and optimal treatment to reduce clinical CV risk.

Obesity-associated dyslipidemia is usually treated with diet and lifestyle interventions. The Expert Panel Guidelines for Cardiovascular Health and Risk Reduction in Children and Adolescents provide evidence based guidance for dietary management of dyslipidemia [46]. The first nutritional approach is based upon the CHILD 1 diet (total fat 30% of daily caloric intake, saturated fat 8–10%, monounsaturated and polyunsaturated fat up to 20%, cholesterol < 300 mg/d, avoid trans fats, encourage high dietary fiber intake from foods). If dyslipidemia persists, the CHILD-2–TG diet is prescribed (total fat 25–30% ≤7% from saturated fat, ∼10% from monounsaturated fat; <200 mg/d of cholesterol; avoid trans fats, encourage high dietary fiber intake). In addition, low simple sugars are replaced with complex carbohydrates, sugar-sweetened beverages are avoided and the weekly frequency dietary fish is increased). The use of plant sterols or stanol esters can be usefull as an adjunct to low cholesterol diet.

However, children with OW/OB may also suffer from familial hypercholesterolemia, which is characterized by the presence of high levels of LDL-C, that may be need pharmacologic treatment in addition to CHILD-2 -diet. Less than 1% of children and adolescents with OB would qualify for statin treatment [50].

#### Panel recommendation

The appropriate age for screening of dyslipidemia in children with OB is 6 years; screening may be anticipated starting from the age of 2 years in the presence of familial or medical conditions at risk of CV disease [17, 46].

### Prediabetes and type 2 diabetes

The prevalence of pre-diabetes in children/adolescents with OB is on average 17% (95% C.I.: 13–22), according to a meta-analysis including both community/school-based and clinic-based representative cohorts of Europeans, Americans, Asians, and Africans, and considering all the three phenotypes of prediabetes: impaired fasting glucose, impaired glucose tolerance and glycosylated hemoglobin (HbA1c) between 5.7 and 6.4% [51]. The heterogeneity is very high, even among cohorts with similar ethnic composition, with a prevalence ranging from 3 to 56% [51]. The prevalence tends to peak among groups of patients from specialized OB centers and/or with a very high average BMI [51–54]. In fact, not only the risk of prediabetes is almost three times higher in youth with OB compared healthy youth, but it also correlates positively with the BMI [51–54]. Type 2 diabetes has a prevalence of 1.3% [95% C.I.: 0.6–2.1], with no study reporting a prevalence above 2%, according to the above-mentioned meta-analysis, which mainly includes adolescent cohorts [51]. The prevalence may be lower (around 0.3%) when both children and adolescents are considered [44, 55]. Some ethnicities (Native Americans, Africans, Latinos, Asians, and Pacific Islanders) are at increased risk, compared to White youth [10, 56]. A recent study performed in Italian children and adolescents with OW/OB demonstrated that prediabetes was detected in 22.8% children under 10 years of age, although at a significantly lower extent than adolescents (27.6%) [52].

According to the American Diabetes Association and the International Society for Pediatric and Adolescent Diabetes, screening for prediabetes and T2D is considered in children with OW starting from the age of 10 years or at pubertal onset in the presence of at least one of the following additional risk factors: maternal history of diabetes or gestational diabetes during the child’s gestation, T2D in a first- or second-degree relative, traditionally high-risk ethnicity, and signs of IR or associated conditions (acanthosis nigricans, HTN, dyslipidemia, polycystic ovary syndrome (PCOS), or small-for-gestational-age birth weight) [16, 57]. Fasting plasma glucose, 2-h plasma glucose during an oral glucose tolerance test and HbA1c can be used for the screening [58]. In those with normal screening results, screening should be repeated after three years, or before, in case of worsening BMI or strong family history of T2D [16, 57].

Although the prediabetes is not considered a disease but a condition at risk for T2D, the treatment of prediabetes is fundamental to avoid the stress of beta-cell function. Therefore, in youths with prediabetes all guidelines recommend a healthy lifestyle: diet consistent with recommendations and requirements for age and BMI, replacement of juices and soft drinks with water, and regular physical activity (PA) (1 h daily of moderate/vigorous PA, with bone and muscle training at least three times a week). Type 2 diabetes is treated with the above-mentioned lifestyle plus drug therapy tailored to the severity of the metabolic impairment [57]. The HbA1c target is < 7%. At diagnosis, metformin (titrated to 2 g/die) is used alone (HbA1c < 8.5%) or in combination with s.c. basal insulin 0.25–0.5 U/kg/die (HbA1c > 8.5%), preceded by i.v. insulin therapy in case of ketosis, ketoacidosis, or hyperosmolar state. In 90% of cases, the patients on metformin + insulin therapy can be weaned off insulin in 2–6 weeks. If HbA1c does not decrease below 7%, a glucagon-like peptide-1 agonist licensed for use in children can be added to metformin [57]. Patients keeping a high HbA1c (> 9%) need s.c. insulin besides maximal doses of metformin and glucagon-like peptide 1agonist/other medication [57]. Pre-prandial rapid insulin could be necessary. Frequent self-monitoring of blood glucose is needed for patients using insulin or sulfonylurea [17]. HbA1c should be dosed every three months. Retinopathy and albuminuria should be screened at diagnosis and annually [57].

#### Panel recommendation

Youths with OB should undergo a fasting glucose test and HbA1c assessment starting from age 6 years [17]. OGTT and HbA1c should be performed in children with OW/OB from age 10 years or at pubertal onset in the presence of additional risk factors.

### Metabolic syndrome

Metabolic syndrome (MetS) is defined by a cluster of several CMRFs, specifically visceral OB, high BP, dyslipidemia, and altered glucose metabolism, which increase the risk of developing future CV disease and T2D in adulthood [59–62]. Identifying children with MetS might allow pediatricians to promptly identify and treat children with an adverse metabolic phenotype and to prevent future adverse outcomes [62]. However, there are several concerns that limit the use of MetS definition in children and adolescents. First, at least 40 different definitions of pediatric MetS have been proposed [61]. Mainly, they are derived from those used for adults, by applying the age-related cut-off values specific for each component [63]. In turn, this may lead to an over-estimation of diagnosis of MetS in childhood. Moreover, the cut-off values for each component of MetS are frequently derived from reference values that are not specific for the patient’s nationality/ethnicity [61]. Therefore, it is difficult to establish a real prevalence of this condition and to precisely define its clinical implication in youths with OW/OB [59–62, 64] Literature data provided a wide range of prevalence of Mets in pediatric age (from 24.1 to 56.3%) with 3 times higher frequency in males than in females [65, 66]. Therefore, without an international diagnostic criterium the definition of MetS is poorly applicable in the clinical setting [60, 62] and its implications for clinical practice are questionable [66].

The lack of consensus regarding the definition of pediatric MetS is partially related to improvement in our knowledge on the mechanisms underlying the stability of metabolic biomarkers during growth and pubertal development. Differently from adults, in whom gender, ethnicity and body composition are the main factors influencing the pathophysiological basis and main features of MetS, growth and puberty represent additional key factors that need to be taken into consideration.

First, the abnormalities behind MetS develop progressively, following age-related changes linked to OB, so that MetS cannot be diagnosed before the age of 6 years [67]. Second, CMRFs may deteriorate during mid-puberty and improve at late puberty, owing to the physiological changes in IR [68]. Such developmental changes affect the long-term reproducibility of the diagnosis of MetS, thus reducing its predictivity in childhood [69]. Indeed, a large proportion of individuals affected by Mets in childhood no longer meet criteria for Mets during both short- and long-term follow-up [70].

As suggested by the AAP it would be more convenient from a clinical point of view to focus on each of the CMRFs, many of which cluster together and are associated with OB, rather than trying to define MetS [71]. Indeed, a recent large prospective study on 38,589 participants from the International Childhood Cardiovascular Cohort (i3C) Consortium, demonstrated strong association between traditional risk factors (BMI, cholesterol, TG, systolic BP, and youth smoking) from childhood to adulthood, individually and in combination, with the development of incident adult CV events beginning as early as 40 years of age [9]. It should be underlined that for any CMRF the risk lies on a continuum. On this basis, several risk scores, such as the Continuous Metabolic Syndrome Score, have been developed to assess CMR in adolescents. Most of them included a measure of adiposity, and use an internal cohort as reference to create Z-scores. The lack of a standard set of CMRFs represents a strong limitation in its implementation in the clinical setting [72].

#### Panel recommendation

Diagnosis of MetS should be abandoned and treatment of CMRFs should focus on established risk factors. In addition, the detection of CMRFs in children should be reassessed at the end of puberty to confirm the persistence of risk and undergo intensive treatment approaches.

Furthermore, it is necessary to increase awareness on other comorbidities associated to OB and not included in the definition of MetS, such as FLD, polycystic ovary syndrome (PCOS), or obstructive sleep apnea (OSA), which might expose youths with OW/OB to an increased risk for CV disease.

### Fatty liver

The build-up of excess fat in the liver is a common trait of childhood OB. Indeed, this condition, formerly termed NAFLD, has become the leading pediatric chronic liver disease with an estimated general prevalence around 7.4% and up to 52.5% in children with OB [73]. Although partly elucidated, the pathophysiology involves the interplay of genetics (variants in *Patatin-like phospholipase containing domain 3* (*PNPLA3)*, *Transmembrane 6 superfamily member 2 (TM6SF2)*,* and Glucokinase Regulator (GCKR)* genes) and environment (diet, gut microbiota, hormones, and chemicals) [74, 75]. IR acts as cornerstone in its dangerous relationship with visceral adiposity and metabolic dysregulation, as highlighted by the thoughtful terminology transition from the “metabolic (dysfunction)-associated fatty liver disease” [75] to the latest “metabolic dysfunction-associated steatotic liver disease” (MASLD), as proposed by a multi-society Delphi consensus statement [76]. According to this statement, MASLD is diagnosed in children with liver steatosis plus one of the five metabolic abnormalities among youths with OW/OB or WC > 95th percentile, prediabetes, high BP, high plasma TG or low HDL-C. Estimates of IR such as HOMA-IR come to be clinically valuable in patients with suspected MASLD who does not still meet any of the 5 diagnostic criteria [76]. Noteworthy, nearly one-fourth of children with MASLD might rapidly progress to steatohepatitis [73, 77] and the disease course in childhood has been found to be more severe than in adults [76, 77]. Despite the shortage of large longitudinal studies, a long-term cardiometabolic burden with increased morbidity and mortality in young adulthood has been observed for pediatric MASLD [74].

Considering the progressive but often asymptomatic MASLD course [74, 77], screening programs are fundamental. The North American Society of Pediatric Gastroenterology, Hepatology and Nutrition guidelines recommend serum alanine transaminase measurement as first line screening in children with OB, indicating the values > 22 IU/L for girls and > 26 IU/L for boys as cut-offs to define high values [14]. Additional risk factors to be taken into account include family history, Hispanic ethnicity, IR, prediabetes and T2D, central adiposity, dyslipidaemia, PCOS, OSA, hypothyroidism, and panhypopituitarism [10, 14]. MASLD screening should be performed between ages 9–11 years, but it can be done earlier in younger patients with OB [14]. Recent studies from the Italian Society for Pediatric Endocrinology and Diabetology study group on childhood obesity demonstrate that high levels of uric acid and/or reduced glomerular filtration rate are robustly suggestive of MASLD [78]. Therefore, these markers of disease may represent a useful tool for deciding to anticipate the screening of MASLD by using abdominal ultrasound.

Despite low diagnostic accuracy, abdominal ultrasound represents the most widely used imaging method since its feasibility and non-invasiveness [14]. It has been largely recommended as second line screening [14]. Magnetic resonance spectroscopy and controlled attenuation parameters using vibration-controlled transient elastography are able to provide semi-quantitative information on fat liver content, being also informative of fibrosis [79]. Its use is limited because of cost, lack of availability, and of validated cut-offs to determine steatotic liver.

Although liver biopsy currently remains the diagnostic gold standard for fatty liver, being able to detect necro-inflammation and fibrosis, its use is limited by ethical issues and invasiveness [14, 80]. Several non-invasive scores have emerged in this context [14, 80, 81]. Both fatty liver index (FLI) and Hepamet Fibrosis Score (HFS) represent non-invasive scores for diagnosing fatty liver and staging fibrosis, respectively [81]. These scores have been created by using combination of different laboratory tests with demographic features and validated mainly in adult populations [81]. Studies on adult patients demonstrated the efficiency and accuracy of FLI in identifying fatty liver, showing also a significant association with cardiometabolic risk factors [80, 81].

HFS represents one of the most recent and promising non-invasive fibrosis scores [82]. In adult patients with fatty liver, it showed a better performance in diagnosing and staging liver fibrosis compared to Fibrosis-4 (FIB-4) and NAFLD Fibrosis Score (NFS) as the most widely used non-invasive tools for screening of advanced fibrosis in these patients [82].

However, there is a lack of validation for these scores in the pediatric population that currently prevent their use as diagnostic tools [83, 84].

Lifestyle interventions (e.g. dietary changes with avoidance of sugar-sweetened beverages, moderate to high-intensity PA, and limiting screen time activities to < 2 h per day) are still the mainstay of the treatment [14, 77]. Dietary supplements (i.e. vitamin E, omega-3 fatty acids etc.), pro/prebiotics, and insulin sensitizer have produced contrasting results [77, 85]. No licensed drugs are available yet, but with a variety of upcoming and promising agents to reduce steatosis (i.e. Glucagon-like peptide-1 receptor agonists and sodium-glucose cotransporter-2 inhibitors), steatohepatitis (i.e. obeticholic acid), and fibrosis, clear recommendations for MASLD trials are urgently needed [10]. Bariatric surgery is considered in selected cases based on judgment of the multidisciplinary team [77, 85].

#### Panel recommendation

Children or adolescents with OW/OB should be considered at risk for MASLD starting from the age of 6 years in the presence of elevated serum alanine transaminase and other CMRFs and should undergo abdominal ultrasound. The presence of hepatic steatosis should be monitored every 12 months to evaluate the evolution towards fibrosis.

### Polycystic ovary syndrome

Polycystic ovary syndrome (PCOS) is a condition characterized by amenorrhea, oligomenorrhea, or abnormal uterine bleeding, in addition to clinical (acne, hirsutism) and/or biochemical (high testosterone and androstenedione concentrations) evidence of hyperandrogenemia. The overall prevalence of PCOS in adolescents varies from 6 to 18%, depending upon the diagnostic criteria used. PCOS is frequently associated with IR, family history of hyperandrogenemia and/or abnormal glucose metabolism underlying possible oligogenic or polygenic inheritance [86, 87].

Whether PCOS represents a risk factor for CV disease remains unclear in adolescence, where studies are scanty. A systematic review of 23 studies [88], of which at least one third on young adults, concluded that women with PCOS had an increased risk of T2D, HTN, dyslipidemia, cerebrovascular disease, but not coronary disease events, whereas a more recent retrospective study in 174.660 young women with PCOS reported an increased incidence of myocardial infarction, angina, and revascularization: of note, weight gain, and prior T2D were identified as modifiable risk factors, as they predicted progression into CV disease [89]. NAFLD has also been found to be more prevalent and severe and to have an earlier onset in women with PCOS [90]. Pathophysiology of OB, NAFLD and PCOS overlaps in several aspects. Hyperinsulinemia, low grade inflammation, and dysregulated adipokines typical of PCOS and NAFLD are exacerbated by OB [89, 90]. MetS in PCOS has been more recently shown to be significantly associated with the lipid accumulation product calculated from the WC and TG levels in normal-weight and OW women with PCOS but not in the obese women [91]. This might be in line with the observation that PCOS is mostly driven by ectopic fat, known to be associated with reduced insulin sensitivity with lipids playing a well-known role in the development of subsequent MetS [85, 91].

Awaiting for effective personalized approaches, and further understanding from ongoing research, a detailed and well guided medical history is essential to address the diagnosis, follow-up, and management of PCOS. A medical history of PCOS in the mother, intrauterine growth retardation, premature adrenarche, IR, dyslipidemia, and mostly increased WC in the adolescent girl, should be considered as risk factors of CMR associated with PCOS. Regular monitoring of glucose, insulin, and lipids as well as screening for NAFLD should be suggested [92].

The mainstay for treatment remains the implementation of educational, and multidisciplinary interventions aiming at lifestyle changes [92, 93]. Several dietary strategies may be considered to reduce energy intake by approximately 30% compared to estimated individual needs, allowing for weight loss [93]. Moreover, recent guidelines recommend 60 min of moderate to vigorous physical activity three times a week [92]. Lifestyle changes and weight loss exert beneficial effects mitigating hormonal and metabolic alterations (especially IR and hyperandrogenemia), and any liver alterations [92].

In addition to lifestyle changes, pharmacotherapy is required to mitigate clinical and biochemical hyperandrogenism, and menstrual irregularities [92–95]. The gold standard treatment for hyperandrogenism is yet represented by combined oral contraceptive pills, containing a natural estrogen associated with dienogest, drospirenone or a neutral progestin based upon individual characteristics and/or risk factors [92, 94, 95]. Alternatively, low dose ethinyl estradiol could be prescribed. Moderate-to-severe hirsutism requires treatment with combined oral contraceptive pills or an antiandrogen (e.g. spironolactone, flutamide, finasteride) for at least 6–9 months [92] Despite being off-label, metformin could be considered in association with the combined oral contraceptive pill, when desired goals have not been achieved [92].

#### Panel recommendation

Female adolescents with OB and PCOS may represent a population with a high prevalence of CMRFs. Lifestyle changes should be a fundamental part of treatment aimed to improve metabolic and CV risk, in association with the management of classical signs and symptoms of PCOS.

### Obstructive sleep apnea

Obstructive sleep apnea (OSA) is characterized by a complete or partial upper airway obstruction during sleep that causes sleep disruption, intermittent hypoxemia, and increased inflammation. The prevalence of OSA is higher in children and adolescents with OB (13-59%) compared to otherwise healthy children (1–4%) [96]. In addition, youths with OB display a higher risk for more severe OSA compared to non-affected peers (OR 7.6,95% C.I. 6.1–9.1) [97].

Several studies have reported an independent association between OSA and the risk of CM derangement in adults. However, evidence in pediatric age is poor [98, 99]. OSA and OB exert similar pathophysiologic effects on CMR via increased inflammation, oxidative stress, IR, and sympathetic activity. Therefore, a bidirectional nature has been hypothesized in the OSA-OB association. In fact, not only OB heightens the risk for OSA, but also sleep apnea induces weight gain and CMR. Studies investigating the association between OSA and altered glucose metabolism gave contrasting results. Despite a cross-sectional study showed that youths with daytime sleepiness presented higher OGTT 2-hour plasma glucose and HbA1c, high OSA risk was not associated with elevated plasma glucose or HbA1c [98]. A cut-off of apnea-hypopnea index (AHI) > 4.9 events/hour has been reported to predict moderate to severe IR in children with OB independent of general and abdominal adiposity [100]. Similarly, not all studies have consistently demonstrated a relationship between OSA, pediatric OB, and CMRFs, as represented by HTN [99, 101] or cardiac remodeling [102] while very few studies addressed the association with arrhythmia or pulmonary HTN [103]. Children with OSA tend to have higher systolic and/or diastolic BP compared with normal weight individuals, but similar prevalence of HTN [104]. However, unlike with obesity-related HTN, an alteration in the circadian rhythm of BP profile and an increase in BP variability is associated with OSA [105]. A cross sectional study reported that OSAS was associated with 4 times greater odds of LVH, even after adjusting for age, sex, race, and BMI Z-score. The odds of LVH increased in adolescents with severe OSA (AHI > 10 events/h) [106]. The presence of OSA during childhood has been associated with an increased risk of HTN during adulthood [103].

The suspicion of OSA is based on clinical examination and the presence of symptoms such as snoring, daytime sleepiness, headache, attention deficit and hyperactivity. Additional tests for OSA screening in children and adolescents include validated questionnaires such as the Pediatric Sleep Questionnaire [103]. A score ≥ 0.33 indicates the presence of OSA. However, only overnight polysomnography ensures the differential diagnosis between OSA and primary snoring in pediatric patients [103].

Polysomnography is the gold standard for OSA diagnosis in children, an AHI > 1 is diagnostic of OSA. A 5 > AHI < 10 indicates moderate OSA and an AHI > 10 suggests severe OSA. The use of nocturnal pulse oximetry, tonsillar ultrasound, and daytime nap polysomnography have a low negative and positive predictive value for OSA diagnosis [103].

First-line OSA treatment is adeno-tonsillectomy (AT). Youths with AHI > 5/h should undergo an AT. Nevertheless, patients with OB display higher rates of residual OSA after AT and might need non-invasive ventilation treatment, such as continuous positive airway pressure or bilevel positive airway pressure [107]. The main limit of non-invasive ventilation is children’s adherence, which ranges from 11 to 78%. Higher adherence to continuous positive airway pressure therapy is reported in children with more severe OSA [108]. Moreover, multidisciplinary weight loss intervention should be performed as it reduces OSA severity [109] According to current recommendations, bariatric surgery may be considered in adolescents with severe OB and OSA (AHI > 5) [18, 110]. A careful preoperative assessment and intra-and postoperative management are required in children and adolescents with severe OSA who are exposed to high anesthesia risk. Re-evaluation of OSA should be performed with Pediatric Sleep Questionnaire and eventually overnight polysomnography in case of suspected residual OSA every six months. Considering the high prevalence of sleep disorders in pediatric OB, **Panel recommendation.** Screening of OSA should be performed in youths with snoring, disturbed sleep, daytime sleepiness, headache, attention deficit and hyperactivity using the Pediatric Sleep Questionnaire. Diagnosis should be confirmed by polysomnography.

### Physical inactivity

The World Health Organization recommends that all children and adolescents be engaged daily for at least one hour in moderate-to-vigorous PA; additionally, time spent in sedentary behaviors, should be limited to less than 2 hours a day [111]. Formerly, less than 50% of children and adolescents in Europe comply with these recommendations [112]. Compared to non-obese peers, youths with OB tend to have even lower level of PA. Activity times of children with OB were generally shorter than 30’ daily and less than 2 h weekly, accumulating a high amount of sedentary time during their waking hours (ranging from 65 to 90%) [113].

Low level of physical PA and excessive time spent in sedentary behaviours, associated to poor diet, contribute to the development and maintenance of OB [114]. Youths with OB experienced difficulties to begin and maintain PA programs, since they showed impaired motor skills due to excessive body weight, acquiring negative feelings related to the PA practice and creating a vicious circle in which sedentary habits became predominant [115, 116]. On the contrary, adolescents with OB displaying a more active lifestyle had better health-related quality of life compared to their inactive peers [117].

Inactivity negatively impact on cardiorespiratory or muscular fitness, leading to unfavourable measures of adiposity, lipids, metabolic parameters, and CV risk [118, 119].

Low levels of PA and excessive sedentary time represent distinct risk behaviours contributing to increased adiposity and CMR. Indeed, the worst effect on health, adiposity and fitness was associated to a combination of low PA/high sedentary time compared to high PA/low sedentary behaviours [120]. Prospective studies were able to demonstrate an association of moderate-vigorous PA, but not sedentary behaviour, with clustered CMR score [121], while more studies are needed to demonstrate that low PA in children is associated with subclinical and clinical CV disease in adults.

Considering the negative physical effects of excessive body weight on motor skills and motor control it may be useful to assess perceived difficulties in physical tasks and health related physical fitness prior to engage children into a regular PA program. Exercise interventions should be tailored to the physical and psychological limitations related to OB to improve physical self-esteem, PA pleasure, and long-time PA adherence [115, 122]. Improvement of several cardiometabolic and vascular parameter can be obtained as earlier as 12 weeks [123]. Aerobic or combined exercise (aerobic and resistance) seem the most effective modalities to reduce body fat, control OB and related cardiometabolic complications [124].

#### Panel recommendation

This Panel supports the role of aerobic or combined aerobic and resistance exercise in the treatment of CMRFs associated to OB. Exercise should be scheduled into three weekly sessions and should progressively reach the goal of 60 min each. Sedentary time should be gradually replaced by a similar amount of PA activity to meet at least 60 min/day [18, 111]. Walking, remote PA and exergaming might be considered as supplemental tools for fighting inactivity [122].

### Heart

Abnormal cardiac structure and function are described in youths with OW/OB. LVH is the most frequently reported phenotype, ranging between 36.3 and 46.6% in Italian young people with OW/OB [34, 125]. LVH reflects cardiac adaptation to increased pressure overload. In addition, OB is characterized by high circulating volume, due to plasma expansion and increased hematocrit, contributing to both volume and pressure overload. This hemodynamic overload can result in atrial dilatation and left ventricular (LV) diastolic dysfunction.

Echocardiographic examination is currently adopted in clinical practice as a useful and less expensive method to examine LV geometry and function in youths. Echocardiographic evaluation should be performed with standardized methodology based on international guidelines. Due to the lack of longitudinal studies, the best criterion to define LVH in children is still debated. LV mass (LVM) depends strongly on body size, namely height [126]. However, the relationship between LVM and body size is not geometrically linear, and allometric exponents are necessary to use linear (height) or quadratic (body surface area) measures of body size to normalize LVM.

Several methods have been proposed to align LVM to linear growth, however, the universally recommended method is LVM index (LVMi) calculated as LVM (g)/height (m)^2.7^ [126]. Since LVM depends on age and sex, LVH should be defined by the 95th percentile of LVMi expressed as g/h^2.7^ according to the age quantiles by Khoury et al. [127]. Recently, Chinali et al. proposed an alternative method by which LVM is indexed for (height^2.16^+0.09) proposing a single cut-off of 45 g/m^2.16^ defined as the upper limit of normality for LVM index [128]. Either criterion was recently recommended by the Panel of experts of the ESC [13]. On the contrary, the AAP proposed a conservative cut-off of LVMi ≥ 51 g/h^2.7^, the same that was proposed in adults, while others experts in USA found that a cut-point of LVMI ≥ 38.6 g/m^2.7^ had the best balance between sensitivity and specificity to detect blood pressure values over the 90th percentile [129]. In a European population, Di Bonito et al. demonstrated that using 95th percentile of LVMi [127] is more accurate than the adult cut-point proposed by the AAP to identify LVH in youths with OW/OB and HTN [130].

#### Panel recommendation

In agreement with the ESC consensus document, the definition of LVH in youths with OW/OB should be based using pediatric reference data of LVM index [127, 128] and not on adult cut-point. It also suggests that youths with OW/OB should undergo standard echocardiographic examination in the presence of the following comorbidities: confirmed HTN, chronic renal failure, dyslipidemia, T2D, and MASLD. The identification of LVH represent an important step for the stratification of CV risk.

Strategies to reduce the progression of cardiac damage, particularly LVH or LV dysfunction, include lifestyle changes such as reducing sugar, salt, and lipid intake and increasing PA, all actions that may reduce hemodynamic overload.

### Vessels

Recent studies indicate that early atherosclerotic changes can be found already in childhood [131]. The link between OB and atherosclerosis is complex. Probably low-grade inflammation and oxidative stress impair endothelial production of nitric oxide and stimulate proliferation of endothelial cells, thus leading to thickening of the vessel wall [132, 133]. Indeed, there is evidence that children with OB may firstly exhibit signs of endothelial dysfunction, such as impaired flow-mediated dilation [132] and increased carotid–radial pulse wave velocity [133], and successively morphological changes, represented by increased values of carotid intima-media thickness (cIMT), even in the absence of comorbidities [131].

As changes in arterial function precede the development of atherosclerotic lesions, an increased arterial stiffness may be an early marker of increased CV risk. Nowadays, carotid–radial pulse wave velocity is considered the gold standard to evaluating arterial stiffness. Although the measurement of flow-mediated dilation represents an important pathophysiological element linked to altered vascular reactivity, its use is less common in clinical practice, while the ultrasound measurement of the cIMT is more feasible. cIMT has been identified as an independent predictor of CV events in adults. Indeed, the results of a recent meta-analysis showed that a decrease in the progression of cIMT of only 10 mm per year results in a reduction of the relative CV risk by 0.91 in adults [134]. Although the predictive value of cIMT in youths has not been confirmed in longitudinal studies on CV outcomes, cIMT is a frequently used non-invasive measure of preclinical atherosclerosis in pediatric research. Regarding the translation into clinical practice, several issues limit the assessment of cIMT, such as intra-individual variability in ultrasound techniques that undermines reproducibility and reliability [135]. Moreover, ethnic-specific differences, variations associated to height and BMI [136] and the absence of universally accepted cut-offs in children and adolescents indicate that cIMT as well as measurement of carotid–radial pulse wave velocity and flow-mediated dilation cannot be used yet in clinical practice [137].

Early functional and/or structural preclinical signs of atherosclerosis of the vessels may improve after implementation of several treatment strategies involving weight loss in adults [138]. Mediterranean diet has been shown to be effective in normalizing cIMT in children with OB, when associated with moderate-vigorous PA [139]. Exercise training improved vascular endothelial function and arterial stiffness in youths with OW/OB [140]. The reversibility of non-invasive ultrasound markers of endothelial dysfunction is extremely relevant in the context of prevention.

#### Panel recommendation

Despite the promising research on non-invasive tests of vessel function or morphology as markers of subclinical atherosclerosis, their diagnostic use is not supported in youths with OW/OB in the clinical practice.

### Kidney

As reported in adults, both microalbuminuria and low estimated glomerular filtration rate (eGFR) are considered robust markers of chronic kidney disease (KD) and CV disease. In the last years, KD emerged as an obesity-related comorbidity also in childhood [141, 142]. Indeed, a large prospective study demonstrated that adolescents with OW had a hazard ratio (HR) of 3.00 (95% CI, 2.50–3.60) and those with OB had a HR of 6.89 (95% CI, 5.52–8.59) for all-cause treated of kidney failure during a 25-year follow-up period [143]. This finding was confirmed by a recent systematic review that found an association between either OW or OB with non-diabetic kidney failure [HR: 2.17 (95% CI,1.71–2.74)] [HR: 3.41; 95% CI, 2.42–4.79)], respectively [144].

Obesity-related KD in children is a heterogeneous condition including four categories of microalbuminuria or proteinuria and seven categories of GFR [145] (Table 2). Hyperfiltration is a condition generally accepted (eGFR > 125–135 mL/min/1.73 m^2^), but not codified [145]. Therefore, a wide prevalence of KD in youths with OW/OB has been reported [141–143], that might be attributed to the heterogeneous definition of some phenotypes of kidney impairment (such as hyperfiltration) or to the methods used for eGFR measurement [146]. In addition, a complex interplay between inflammatory and metabolic (e.g., IR pathways [144, 147, 148] leads to early kidney morphological changes (e.g., glomerular enlargements) with a potential progressive course from normal function to chronic KD [141, 147].

**Table 2:** Nomenclature for kidney disease*

Kidney measure	Cut-offs
***Estimated Glomerular filtration rate (eGFR)***	ml/min/1.73 m^2^
Normal eGFR (G1)	≥ 90
Mild reduced eGFR (G2)	< 90 ≥ 60
Moderately Reduced eGFR (G3a)	< 60 ≥ 45
Moderately Reduced eGFR (G3b)	< 45 ≥ 30
Severely reduced GFR (G4)	< 30 ≥ 15
Kidney failure (G5)	< 15
***Albuminuria/Proteinuria***	
Normoalbuminuria/mildly increased (A1)	AER < 30 mg/d; ACR < 30 mg/g (< 3 mg/mmol)
Moderately increased (moderate)(A2)	AER 30–300 mg/day; ACR 30–300 mg/g (3–30 mg/mmol)
Severely increased (severe) (A3)	AER > 300 mg/d; ACR > 300 mg/g (> 30 mg/mmol)
Nephrotic-range/syndrome	AER > 2200 mg/d; ACR > 2200 mg/g (> 220 mg/mmol)

AER: albumin excretion rate. ACR: albumin-creatinine ratio;

*Ref #137

The first approach for KD screening is based on the measurements of microalbuminuria and serum creatinine (SCr). While the method for the evaluation of microalbuminuria is well established, more controversial is the assessment of renal filtration [145, 146]. The gold standard for GFR evaluation is represented by Cystatin-C measurement, that is not influenced by diet, physical activity, or muscle mass. However, the assessment of SCr represents the less expensive and widely adopted method to evaluate glomerular function in clinical practice [146]. The enzymatic method should be preferred to the Jaffè’s colorimetric method. To evaluate eGFR, several creatinine-based equations that use correction factors such as height or age have been proposed [see Additional file 1] [146]. However, an international consensus about the best formula is still lacking in youths. Recently, two studies demonstrated that eGFR Full Age Spectrum (eGFRFAS) based on age- or height is more effective than other equations in youths with OW/OB to identify individuals with abnormal CMR associated with mild reduced eGFR [149, 150].

Since OB represents a modifiable risk factor for KD, weight loss strategies (including dietary changes, salt intake reduction and increased PA) are the first-line treatment [141, 142, 147]. A strict control of BP is necessary to maintain BP levels < 90th percentile. On the other hand, the evidence of a pharmacological approach in children with OB and KD is still limited and controversial [141, 147, 148]. In addition to the commonly used reno-protective drugs (e.g., angiotensin-converting enzyme inhibitors), glucagon-like peptide 1 receptor agonists in adolescents with OB and KD are promising to protect kidneys through their metabolic and anti-inflammatory effects, but data are still contrasting [141, 147, 148]. Metabolic bariatric surgery has been also found to positively impact on KD in adolescents with severe OB, but its use is limited and long-term effects are still lacking [147, 148].

#### Panel recommendation

Considering the detrimental role of OB on kidney function and its prognostic relevance, microalbuminuria and SCr should be assessed in all children with OW/OB from the age of 6 years at the first evaluation. A careful monitoring of renal function should be considered semi-annually in the presence of microalbuminuria or abnormal values of SCr or every three to six months in the presence of other comorbidities such as HTN, prediabetes/T2D, or PCOS.

**Table 3:** Clinical-based and biochemical screening for cardiometabolic risk factors or comorbidities associated to cardiometabolic risk in children and adolescents with obesity and the relative screening frequency according to age

	*≤* 5 years	6–10 years	> 10 years	Screening frequency	
HTN	Blood pressure from age 3 years	Blood pressure		At every clinic visit	
Dyslipidemia	Fasting lipids from age 2 years in presence of familial or medical conditions at risk of cardiovascular disease	Fasting lipids		• After 3 years (if negative) • More frequently if rapid weight gain or development of other cardiometabolic comorbidities • Every 6–12 months (if abnormal)	
Prediabetes, T2D	-	Fasting glucose and HbA1c; OGTT and HbA1c in presence of pubertal onset plus additional risk factors*	OGTT and HbA1c in presence of additional risk factors*	• After 3 years (if negative) • More frequently if rapid weight gain or development of other cardiometabolic comorbidities • Fasting glucose and HbA1c every 3 months (if prediabetes)	
MASLD	-	Liver ultrasonography in presence of elevated serum ALT or other CMRFs		• ALT every 2 years (if negative) • More frequently if rapid weight gain or development of other cardiometabolic comorbidities • 3–6 months (if abnormal) to confirm results and guide subsequent laboratory testing • Ultrasonography every 12 months (steatosis)	
PCOS	-	-	Hormonal assessment in presence of persistent irregular menstrual cycles and clinical and/or biochemical signs of hyperandrogenism**	Clinical-based screening at every clinic visit starting 2 years after menarche	
OSA	Pediatric Sleep Questionnaire in the presence of suggestive symptoms (snoring, disturbed sleep, daytime sleepiness, headache, attention deficit, hyperactivity) Diagnosis confirmed by polysomnography			Clinical-based screening at every clinic visit	
Physical inactivity	Questionnaire on physical activity/ sedentary time			At every clinic visit	
Left ventricular hypertrophy	-	Echocardiography in presence of confirmed HTN		6–12 months if persistent HTN despite treatment, or signs of target organ damage	
Preclinical atherosclerosis	-	Carotid–radial pulse wave velocity, flow-mediated dilation and cIMT measurements are not indicated in clinical practice		Not indicated	
Kidney disease	-	Microalbuminuria and sCr		• Annually (if negative) • Semi-annually in presence of microalbuminuria or abnormal values of SCr • Every 3–6 months in presence of other comorbidities (HTN, prediabetes/T2D, or PCOS)	

Abbreviations: ALT, alanine transaminase; CMRFs, cardiometabolic risk factors; HbA1c, glycosylated hemoglobin A1c; HTN, hypertension; MASLD, metabolic dysfunction-associated steatotic liver disease; OGTT, oral glucose tolerance test; OSA, obstructive sleep apnea; PCOS, polycystic ovary syndrome; sCr, serum creatinine; T2D, type 2 diabetes

*diabetes risk factors: type 2 diabetes in a first- or second-degree relative, high-risk racial/ethnic groups, maternal history of diabetes or gestational diabetes, signs or conditions associated with insulin resistance, such as hypertension, dyslipidemia, polycystic ovary syndrome, acanthosis nigricans, or small for gestational age birth weight

** total and free testosterone, 17-hydroxyprogesterone, androstenedione, free thyroxine, thyroid stimulating hormone, luteinising hormone, follicle stimulating hormone, and prolactin

## Conclusions

The growing evidence base suggests OB to be a major contributor to CVD via direct and indirect mechanisms. Consequently, there is a pressing need for effective strategies to monitor obesity-related comorbidities in children and adolescents, aiming to reduce the burden of CV morbidity and premature mortality within this demographic.

This-position paper, drawing upon concepts and perspectives outlined in a comprehensive document by the “Childhood Obesity study group” of the Italian Society for Pediatric Endocrinology and Diabetology, focuses on multidimensional and integrated interventions aimed at mitigating or reducing the impact of CV and metabolic disorders in pediatric patients with OB. Four are the main recommendations (for action): *a).* early detection of OB comorbidities, including HTN, dyslipidemia, prediabetes or T2D, MASLD, polycystic ovary syndrome, obstructive sleep apnea, decline in kidney function and inactivity by a comprehensive assessment in youths with OB; *b).* treatment of OB for promoting weight loss, which is associated with a reduction of all CVRFs; *c).* specific treatment of comorbidities, through lifestyle modifications or subsequent pharmacological treatment in conjunction with lifestyle for suitable individuals; *d).* monitoring comorbidities for mitigating future morbidity and mortality. We summarized in Table 3 the clinical-based or biochemical screening tools for cardiometabolic risk factors and comorbidities associated to cardiometabolic risk in children and adolescents with OB and the relative screening frequency according to age.

Available evidence strongly suggests early intervention on OB comorbidities although longitudinal studies are necessary to quantify its efficacy on morbidity and mortality and to suggest improvements in the strategy and procedures of the interventions themselves.

## Electronic supplementary material

Below is the link to the electronic supplementary material.

Supplementary Material 1

Supplementary Material 2

## Data Availability

Not applicable.

## Abbreviations

AAP: American Academy of Pediatrics
AHI: apnea-hypopnea index
BMI: body mass index
BP: blood pressure
CMRFs: cardiometabolic risk factors
CV: cardiovascular
eGFR: estimated glomerular filtration rate
eGFRFAS: estimated glomerular filtration rate Full Age Spectrum
ESC: European Society of Cardiology
FLD: fatty liver disease
HbA1c: glycosylated hemoglobin A1c
HR: hazard ratio
HTN: hypertension
IR: insulin resistance
KD: chronic kidney disease
LDL-C: low density lipoprotein-cholesterol
LV: left ventricular
LVH: left ventricular hypertrophy
LVM: left ventricular mass
MASLD: metabolic dysfunction-associated steatotic liver disease
MetS: metabolic syndrome
NAFLD: non-alcoholic fatty liver disease
Non-HDL-C: non-high-density cholesterol
OB: obesity
OSA: obstructive sleep apnea
OW: overweight
PA: physical activity
PCOS: polycystic ovary syndrome
SCr: serum creatinine
TG: triglycerides
T2D: type 2 diabetes
WC: waist circumference
WHtR: waist-to-height ratio
