# Supplementary material for: Cardiometabolic risk in children and adolescents with obesity: a position paper of the Italian Society for Pediatric Endocrinology and Diabetology
Source: Ital J Pediatr. 2024 Oct 8;50:205. doi: 10.1186/s13052-024-01767-x (PMC11463079; doi:10.1186/s13052-024-01767-x)
Supplement: Supplementary file 1 — Supplementary Material 1 [file 13052_2024_1767_MOESM1_ESM.docx]

**Table 1**. Equations for calculation of estimated glomerular filtration rate.

| **Denomination** | **Equation** |
| --- | --- |
| eGFR_Schwartz_ ^a^ | 0.413 x height (cm)/Scr (mg/dL) |
| eGFR_Schwartz-Lyon_ ^b^ | 0.368 x height (cm)/Scr (mg/dL). 0.413 x height (cm)/Scr (mg/dL) in boys aged >13 years. |
| eGFRFAS_age_^c^ | 107.3/[(Scr(mg/dL)/Q_age_)].  Q_age_= 0.21+0.057 x Age - 0.0075 x Age^2^ + 0.00064 x Age^3^ - 0.000016 3 Age^4^ for boys  Q_age_= 0.23+0.034 x Age - 0.0018 x Age^2^ + 0.00017 x Age^3^ - 0.0000051 x Age^4^ for girls |
| eGFR_SchwartzCysC_^d^ | 40.9 x (1.8/CysC)^0.931^ (CysC in mg/L) |

Scr: serum creatinine. CysC: serum Cystatin C.

1. Schwartz GJ, Munoz A, Schneider MF, et al. New equations to estimate GFR in children with CKD. J Am Soc Nephrol 2009;20:629–637
2. Selistre L, De Souza V, Cochat P, et al. GFR estimation in adolescents and young adults. J Am Soc Nephrol. 2012;23:989–996
3. Pottel H, Hoste L, Martens F. A simple height-independent equation for estimating glomerular filtration rate in children. Pediatr Nephrol 2012; 27:973–979
4. Schwartz GJ, Schneider MF, Maier PS, et al. Improved equations estimating GFR in children with chronic kidney disease using an immunonephelometric determination of cystatin C. Kidney Int. 2012;82:445-453.
