# Supplementary material for: Cardiometabolic risk in children and adolescents with obesity: a position paper of the Italian Society for Pediatric Endocrinology and Diabetology
Source: Ital J Pediatr. 2024 Oct 8;50:205. doi: 10.1186/s13052-024-01767-x (PMC11463079; doi:10.1186/s13052-024-01767-x)
Supplement: Supplementary file 2 — Supplementary Material 2 [file 13052_2024_1767_MOESM2_ESM.docx]

Napoli, 19^th^ August 2024

Professor Carlo Caffarelli

Italian Journal of Pediatrics

Dear Prof. Caffarelli,

enclosed you will please find the revised manuscript entitled “**Cardiometabolic risk in children and adolescents with obesity: a Position paper of the Italian Society for Pediatric Endocrinology and Diabetology**.”

We thank the Editor and reviewers for their positive comments and the helpful suggestions.

We have replied to all the comments arisen from the reviewers and fulfilled their requests.

We hope that the paper may be now suitable for publication in your journal.

Thank you for your kind consideration.

Prof. Giuliana Valerio

Full Professor of Pediatrics

Department of Medical, movement and wellbeing Sciences

University of Naples Parthenope

Via Medina 40

80133 Naples, Italy

**Dipartimento delle Scienze Mediche, Motorie e del Benessere - DiSMMeB**

Segretario Amministrativo**: Dott. Generoso Gentile**

E-mail: generoso.gentile@uniparthenope.it
